# Mirror Neurons in a New World Monkey, Common Marmoset

**DOI:** 10.3389/fnins.2015.00459

**Published:** 2015-12-10

**Authors:** Wataru Suzuki, Taku Banno, Naohisa Miyakawa, Hiroshi Abe, Naokazu Goda, Noritaka Ichinohe

**Affiliations:** ^1^Department of Ultrastructural Research, National Institute of Neuroscience, National Center of Neurology and PsychiatryTokyo, Japan; ^2^Ichinohe Neural System Group, Lab for Molecular Analysis of Higher Brain Functions, RIKEN Brain Science InstituteSaitama, Japan; ^3^Division of Sensory and Cognitive Information, National Institute for Physiological SciencesAichi, Japan; ^4^Department of Physiological Sciences, The Graduate University for Advanced Studies (Sokendai)Aichi, Japan

**Keywords:** *in vivo* imaging, New World monkey, premotor cortex, primate, superior temporal sulcus

## Abstract

Mirror neurons respond when executing a motor act and when observing others' similar act. So far, mirror neurons have been found only in macaques, humans, and songbirds. To investigate the degree of phylogenetic specialization of mirror neurons during the course of their evolution, we determined whether mirror neurons with similar properties to macaques occur in a New World monkey, the common marmoset (*Callithrix jacchus*). The ventral premotor cortex (PMv), where mirror neurons have been reported in macaques, is difficult to identify in marmosets, since no sulcal landmarks exist in the frontal cortex. We addressed this problem using “*in vivo*” connection imaging methods. That is, we first identified cells responsive to others' grasping action in a clear landmark, the superior temporal sulcus (STS), under anesthesia, and injected fluorescent tracers into the region. By fluorescence stereomicroscopy, we identified clusters of labeled cells in the ventrolateral frontal cortex, which were confirmed to be within the ventrolateral frontal cortex including PMv after sacrifice. We next implanted electrodes into the ventrolateral frontal cortex and STS and recorded single/multi-units under an awake condition. As a result, we found neurons in the ventrolateral frontal cortex with characteristic “mirror” properties quite similar to those in macaques. This finding suggests that mirror neurons occur in a common ancestor of New and Old World monkeys and its common properties are preserved during the course of primate evolution.

## Introduction

Mirror neurons are cells that respond both when performing a motor act (such as grasping, breaking, or tearing) and when observing others performing a similar act. Mirror neurons are suggested to be involved in the understanding of others' action and intention and in imitation (Gallese et al., 1996; Iacoboni et al., 1999; Rizzolatti et al., 2001), by forming a link between the sensory description and the individual motor representation (Rizzolatti and Craighero, 2004), and may also be a neural substrate of language evolution (Rizzolatti and Arbib, 1998). They have been found in the ventral premotor cortex (PMv) and inferior parietal lobule (Rizzolatti and Craighero, 2004; Ferrari et al., 2009). These regions are also shown to be active during the observation of others' action in functional neuroimaging studies (Buccino et al., 2001; Caspers et al., 2010; Nelissen et al., 2011).

Mirror neurons were first discovered in Old World macaque monkeys, *Macaca nemestrina* (di Pellegrino et al., 1992; Gallese et al., 1996; Umiltà et al., 2001; Kohler et al., 2002; Ferrari et al., 2003; Fogassi et al., 2005; Bonini et al., 2010), and subsequently found in *Macaca mulatta* (Tkach et al., 2007; Caggiano et al., 2009, 2011, 2012, 2013; Kraskov et al., 2009; Dushanova and Donoghue, 2010) and humans (Mukamel et al., 2010). So far, the most phylogenetically ancient species found to have mirror neurons are songbirds whose forebrain has auditory-vocal mirror neurons suggested to be involved in imitative vocal learning (Prather et al., 2008; Keller and Hahnloser, 2009; Giret et al., 2014). It is suggested that mirror neurons emerge owing to adaptation through evolution to fulfill particular functions, e.g., understanding what others are doing and social learning including observation learning and imitation learning (Rizzolatti and Craighero, 2004; Bonini and Ferrari, 2011; but see Heyes, 2010). In this study, we showed mirror neurons responsive to grasping action found in Old world monkeys exist in New world monkeys, indicating that the evolution of mirror neurons can be traced to a common ancestor of Old and New world monkeys with primate specific motor repertories such as reaching, grasping, and manipulation actions with hands (Cartmill, 1974; Bloch and Boyer, 2002; Stepniewska et al., 2005). New World monkeys evolutionally separated approximately 15 million years before the split between apes and Old World monkeys (Goodman et al., 1998; Chatterjee et al., 2009). This study would provide valuable insight into our understanding of primate evolution.

In this study, we investigate whether mirror neurons exist in the frontal cortex of common marmosets (*Callithrix jacchus*). Marmosets are highly social animals (Ferrari, 1992; Rothe et al., 1993) and show unique social learning (Voelkl and Huber, 2000, 2007). Because the cortex of common marmosets is lissencephalic (flat), it is more difficult to identify brain areas *in vivo* for neuronal recording in common marmosets than in macaque monkeys that have a clear sulcus landmark for brain area identification. To find the target area efficiently, we combined *in vivo* surface connection imaging with electrophysiology (Ichinohe et al., 2012). We take advantage of the superior temporal sulcus (STS), because it is one of the clear and rare landmark sulci of the species and it contains cells representing others' actions (Suzuki et al., 2015). We simultaneously recorded multiunits from a part of the STS containing cells responsive to the sight of others' action, which was determined by multiunit recording beforehand, and from a circumscribed area in the frontal cortex, which was identified *in vivo* to have cells fluorescently labeled by a retrograde tracer that had been injected to the STS site after the first recording.

## Materials and methods

### Subjects

Experiments were performed with three adult common marmoset monkeys (*C. jacchus;* weighing 300–400 g). This study was approved by the Experimental Animal Committee of the National Institute of Neuroscience and Psychiatry, and the animals were cared for in accordance with the “Guiding Principles of the Care and Use of Animals in the Field of Physiological Science” of the Japanese Physiological Society.

### Electrophysiological recordings under anesthesia

As a general guideline to the preparation of marmosets, we followed Bourne and Rosa's procedure (Bourne and Rosa, 2003). The food was withdrawn in the evening before the day of the experiment. Surgery and electrophysiological recordings were conducted under anesthesia induced by an intramuscular injection of ketamine hydrochloride (Ketalar, 25 mg/kg i.m.) following an intramuscular injection of atropine sulfate (0.15 μg/kg), and maintained with an intravenous infusion of remifentanil (Ultiva, 0.1 μg/kg/min). During the recordings, muscular paralysis was induced with rocronium bromide (Eslax, 13 μg/kg/min). The animal was artificially ventilated with a mixture of 70% N_2_O, 30% O_2_, and, when necessary, 1.0–2.0% isoflurane. The ECGs, expired CO_2_, and rectal temperature were monitored continuously throughout the experiments. The animals were placed in a stereotactic apparatus and the head holder and the recording chamber were implanted on the skull. Before the recordings, the pupil was fully dilated with topical tropicamide (0.5%) and phenylephrine hydrochloride (0.5%). A contact lens whose power was measured using a retinoscope was used to focus the eye contralateral to the recorded hemisphere at a distance of 57 cm.

After craniotomy and duratomy, electrodes were inserted with reference to two sulcal landmarks, STS and the lateral fissure. A micromanipulator lowered a linear array 32-channel multicontact electrode (Neuronexus, Ann Arbor, MI, US) perpendicular to the cortical surface of the posterior and ventral parts of STS, where strong responses to others' grasping action were commonly observed under our anesthetic condition. The linear array multicontact electrode contained four shanks (400 μm shank separation) and each shank had eight contacts (impedance, ~1 MΩ at 1 kHz) with an intercontact spacing of 200 μm. Multiunit activities were simultaneously recorded from the 32 (4 shanks × 8 contacts) contacts. For two animals, only 4–5 bottom contacts of each shank were inserted into the cortex to minimize the penetration damage of the cortex, for the subsequent experiments. The timing of multiunit activity and task events (stimulus onset and offset) were recorded and stored with < 1 ms resolution using a TDT signal processing system (RZ2, Tucker-Davis Technologies, Alachua, FL, US).

The visual stimulus set consisted of 33 movies that showed reaching and grasping motor acts of an actor marmoset. Actions performed by the marmosets were recorded with a video camera (HDR-CX560V, Sony, Tokyo, Japan) at 30 frames per second. Using graphics software (Adobe Premiere Pro CS4, Adobe, San Jose, CA, US), we edited the recorded videos to generate video clips of 1 s duration (30 frames) with a resolution of 640 × 480 pixels. Three different marmoset subjects were used as the actor animal with two different types of food (a piece of potato and bun) and with two different views (frontal and lateral). The size of the stimulus (video clip) was ~20°. Each stimulus was presented 12 times in a pseudorandom order.

### Fluorescent *in vivo* surface connection imaging and implantation of chronic electrodes

CTB-Alexa555 (Invitrogen-Molecular Probes, Eugene, OR, US) was used as a retrograde tracer. The tracer was diluted to 1% in 0.1 M phosphate-buffered saline (PBS), and 0.12–0.15 μL of the tracer solution was pressure-injected through a glass micropipette with a 50-μm-inner-diameter tip, which was attached to a 10 μL Hamilton syringe. The injection sites were immediately confirmed under a fluorescence stereomicroscope (VB-G05, Keyence Corporation, Osaka, Japan) with a filter for red fluorescent protein (RFP, emission, 540/25; absorption, 572).

The retrograde tracer was injected into an area in STS that contained the cells strongly responsive to the video clip of others' action, as determined from the electrophysiological recording under anesthesia (see above). For all the animals, the injection site was close to the posterior tip of STS and ventral to STS. After the injection, an artificial dura was placed on the cortex and the bone was put back and the wound was closed. All surgical procedures were the same as in the electrophysiological experiment except that remifentanil and rocronium bromide were not administered. The anesthesia was maintained using a mixture of 70% N_2_O, 30% O_2_, and 2.0% isoflurane.

One to three weeks after the injection, craniotomy and duratomy around STS and the frontal regions were performed. Fluorescently labeled spots were identified *in vivo* in the lateral frontal cortex and at the injection site as well under a fluorescence stereomicroscope with a filter for RFP (Ichinohe et al., 2012). The labeled spots were observed in the lateral frontal cortex including area 6V according to a histological examination, which will be explained later. Two micromanipulators were used to lower linear array multicontact electrodes (Neuronexus, Ann Arbor, MI, US) vertically perpendicular to the cortical surface in the STS injection site and in the labeled spots in the lateral frontal cortex for the chronic experiment. The number of shanks, impedance, and intercontact spacing were the same as those used for the experiment conducted under the anesthetic condition.

### Electrophysiological recordings from awake animals

After the animal recovered from the electrode implantation surgery (about 2 days), we conducted multiunit recording. During the experiments, the animal sat comfortably in a primate chair and the head was fixed. Multiunit activity was recorded simultaneously from STS and the ventrolateral frontal cortex. However, the signals from the electrode implanted in STS of the second and third animals were not detected, probably owing to damage caused by several penetrations (electrodes for recordings under the anesthetic and awake conditions and a glass pipet for tracer injection). When an event (e.g., touching the food by the experimenter or the animal) occurred, the experimenter pushed a button to turn on a small LED. The button signaled to the TDT system. The LED was invisible to the animal but visible to a video camera (HDR-CX560V, Sony, Tokyo, Japan, 30 frames per seconds). The animal behaviors recorded by the video camera and neuronal data were synchronized with reference to the LED state accompanied by each behavioral event. This was analyzed offline frame by frame. Thus, the time resolution of this synchronization was 33.3 ms, which was limited by that of the video camera.

We recorded visual responses of cells in STS and the ventrolateral frontal cortex while an experimenter was reaching and grasping food in front of the animal with its head fixed. Food was put on a tray, which was placed just in front of the primate chair. The position of the food on the tray was the same across all trials in a session. The experimenter performed the following grasping action types. He/she reached and grasped (1) a piece of banana with his/her hand, (2) a piece of bun with his/her hand, (3) a piece of banana with a pair of forceps, and (4) he/she mimed to reach and grasp as if there was a piece of food. These four action types were performed from either the right or left side of the animal (in total, 4 action types × 2 reaching directions = 8 conditions). Moreover, we recorded motor-related responses of cells while the animal took a piece of banana/bun from a tray with its head fixed. Each condition was designed as a block that consisted of at least 10 trials and each block was pseudorandomly intermingled.

### Data analysis

The neuronal responses, expressed as the mean firing rate (spikes per second) of multiunit activity, were measured in two different time epochs: Epoch 1 corresponds to a 1-s period centered at the hand-food contact and Epoch 2 corresponds to a 1-s period starting 5 s before the hand-food contact (baseline response). The significance of the response when the animal was observing grasping action executed by the experimenter and when the animal itself was grasping was examined by comparing the responses in Epoch 1 with those in Epoch 2 by the paired *t*-test. Individual multiunits were defined as mirror neurons when the neuronal responses were significant while observing at least one of the eight grasping action conditions [*p* < 0.05 after Bonferroni correction for multiple comparisons (*p* < 0.05/8)] and while executing grasping action (*p* < 0.05). To analyze the effect of the grasping action types under the observation conditions, we applied Two-way factorial ANOVA (with reaching direction and grasping action type as factors) to the neuronal responses during Epoch 1. The time course of the population neuronal responses for the mirror neurons responsive during observation (execution) was calculated by aligning at the moment when the experimenter's hand touched the food under the most preferred grasping action condition (animal's hand touched the food), normalizing by the maximum magnitude of response during the grasping action, and averaging over all the mirror neurons.

We also analyzed the multiunits that responded while observing under at least one of the eight grasping action conditions [paired *t*-test between Epochs 1 and 2, *p* < 0.05 after Bonferroni correction for multiple comparison (*p* < 0.05/8)], but failed to respond while executing a grasping action. The time course of the population activity was calculated by aligning at the moment when the experimenter's hand touched the food for each grasping action type in the preferred reaching direction for each multiunit and normalized by its maximum response, and then averaged over the multiunits. Two-way factorial ANOVA (with recording area and grasping type as factors) was applied to the responses around the time the food was touched (Epoch 1). We also analyzed normalized responses by calculating z scores and obtained similar results.

To confirm that single units showed the mirror neuron properties, single-unit data were sorted offline using the t-distribution E-M sorting algorithm provided in the Plexon Off-line Sorter software from the multiunit data (Plexon Inc., Dallas, Texas, US).

### Histological processing

After all the experiments, the animals were sedated with ketamine hydrochloride (Ketalar, 25 mg/kg i.m.) and overdosed with sodium pentobarbital (Nembutal, 75 mg/kg i.p.). The animals were perfused intracardially, in sequence, with 0.1 M PBS (pH 7.4) and 4% paraformaldehyde in PBS (Merck, Whitehouse Station, NY, US), and a brain block was put in ice-cold 0.1 M PBS with 10, 20, or 30% sucrose. Coronal sections were prepared at 50 μm in a series of three sections.

We cut a 50-μm-thick section coronally with a freezing microtome (Yamato-Koki, Saitama, Japan). We divided the section into three. One in three consecutive sections was used for immunoperoxidase staining of CTB-Alexa555. Sections were blocked in PBS-TG for 1 h at room temperature and subsequently incubated with an Alexa555-conjugated anti-rabbit antibody (1:1000; Invitrogen-Molecular Probes) in PBS-TG for 2 days at 4°C. After washing with 0.1 M PBS, the sections were incubated with biotinylated anti-rabbit polyclonal goat antibody (1:200; Vector Laboratories Inc., Burlingame, CA, US) for 1.5 h at room temperature. Immunoreactivity was visualized using an ABC Elite kit (Vector Laboratories Inc.), followed by diaminobenzidine histochemistry with 0.03% nickel ammonium sulfate. All the sections were mounted on gelatin-coated glass slides, air dried, dehydrated in graded EtOH solutions, immersed in xylene, and cover-slipped in DPX (Sigma-Aldrich Co., Buchs, Switzerland). For areal demarcation, second sections in the three series were stained for myelin (Pistorio et al., 2006), and the third sections for Nissl substrate with thionin. In the third animal, we failed to stain the first sections in three series by immuno-histochemistry of CTB due to unknown reason. However, we confirmed that linear array multicontact electrodes were implanted in the ventrolateral frontal cortex from sections stained by Nissl substrate and myelin.

**Figure 1:**
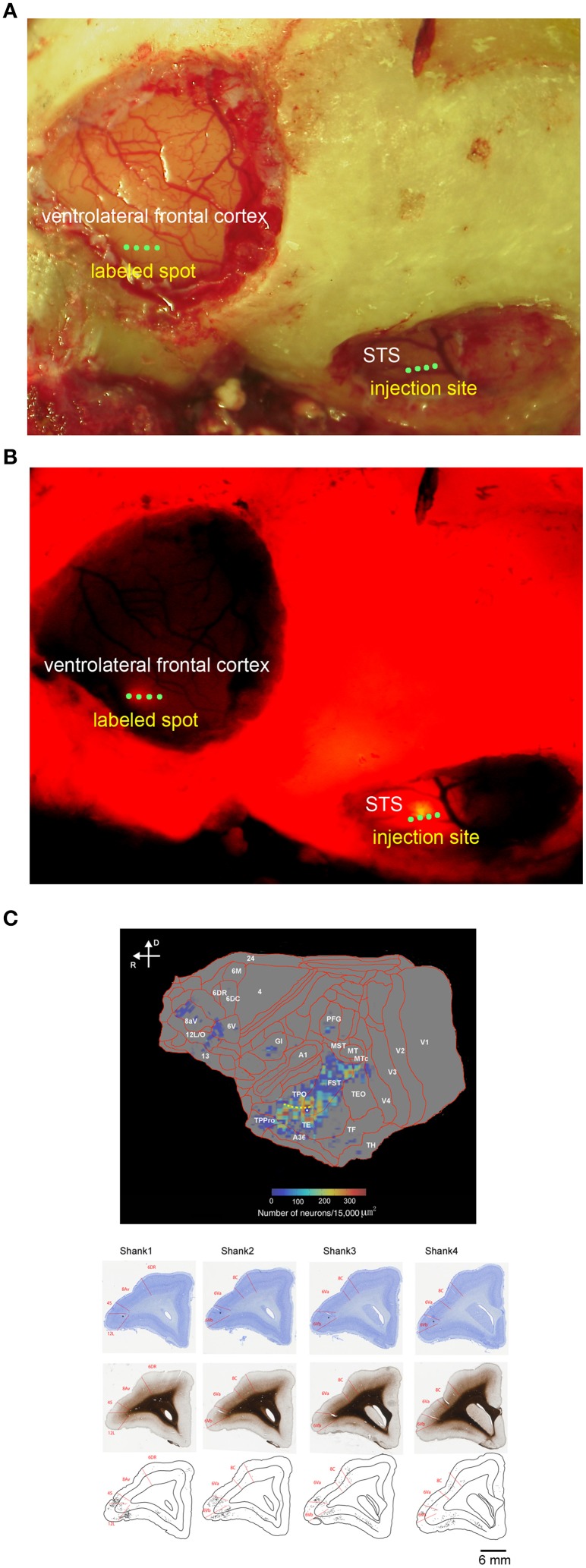
*****In vivo*** surface connection imaging and flat map of CTB-Alexa555 staining**. **(A)** Cortical surface of the lateral frontal cortex and temporal cortex of a common marmoset after craniotomy and duratomy. Four green dots in the ventrolateral frontal cortex and STS indicate the implantation position of four shanks of linear array multicontact (32-channels) electrodes. **(B)** *In vivo* surface connection imaging of the lateral frontal cortex and temporal cortex shown in **(A)**. CTB-Alexa555 was injected into the posterior part of STS, as determined by electrophysiological mapping. The image was adjusted for brightness and contrast for presentation purposes. **(C)** Top: Two-dimensional “unfolded” labeled cell density map of the cortical surface, constructed by computer graphic reconstructions of the cortex prepared with the software program CARET (Van Essen et al., 2001). Pseudocolor represents the density of labeled cells. The yellow dotted line indicates STS. Bottom: Coronal sections stained by Nissl substrate and myelin showing the areal boarder in the ventrolateral frontal cortex and distribution of the labeled cells. The asterisks indicate recording tracks of shanks of electrodes in the ventrolateral frontal cortex. D, dorsal; R, rostral.

The distribution of retrogradely labeled cells was analyzed and plotted for every 300 μm. The materials were analyzed under a Nikon Eclipse E-800 microscope (Nikon Co., Tokyo, Japan) at 100X, 200X, and 400X magnifications. The cells were plotted and the outer surface of the cortex, the white matter, and the middle of layer 4 were drawn using Neurolucida (MBF Bioscience, Williston, VT, US) through a MicroFIRE digital camera (MicroFire Technology Company, Ltd., Shenzhen, China) attached to the microscope. To determine the density of retrograde neurons, we used a program that operates on Matlab (Mathworks, Natick, MA, USA) (a gift from Dr. Eiji Hoshi). This program allowed us to load and display the digitized data from each section, to place landmarks on the displayed section, and to draw a line onto which labeled neurons were projected. It further allowed us to align the positions of the labeled neurons with landmarks in multiple sections. Using this program, we drew a curved line on each coronal section. In combination with this program and software of CARET by van Esssen lab (http://brainvis.wustl.edu/wiki/index.php/Caret:About), we drew a flat map with a heat map for the retrogradely labeled neurons (Figure 1C).

## Results

**Figure 2:**
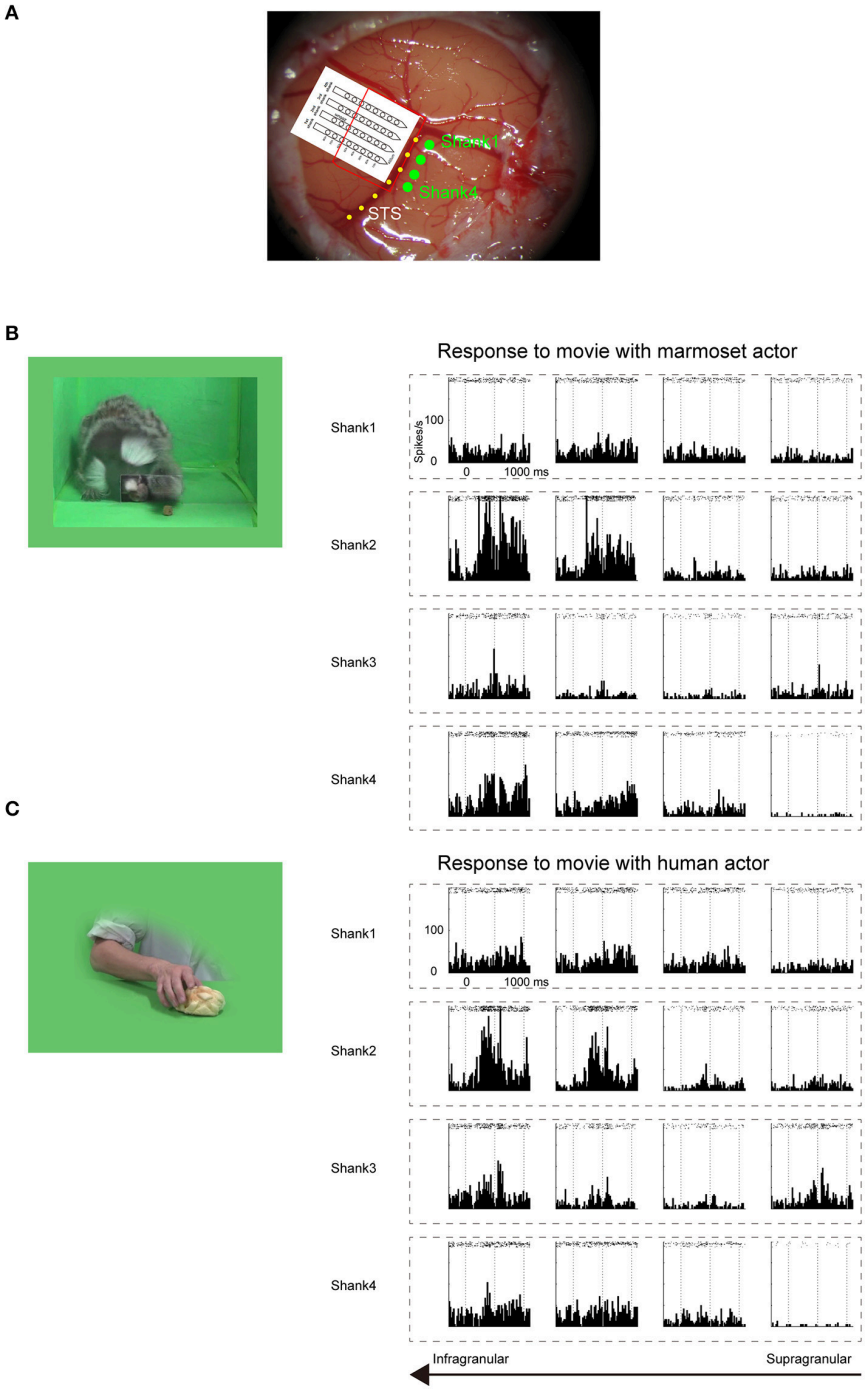
**Electrophysiological mapping of STS under anesthetic condition**. **(A)** Cortical surface around STS of a common marmoset. Four green dots indicate the penetration sites of four shanks of a linear array multicontact electrode. The red line indicates the presumed cortical surface because only 4–5 bottom contacts of each shank were inserted. The yellow dotted line indicates STS. **(B)** Multiunit responses to the sight of grasping action of another marmoset, which were arranged by channel configuration. Rasters and peristimulus time histograms were aligned to the stimulus onset at time = 0. The bin width for the peristimulus time histogram was 20 ms. To minimize damage to the cortex caused by the electrode penetration, multiunits were recorded only from channels on the upper part of each shank. The multiunits on the bottom two channels on the second shank strongly responded to the movie. **(C)** Multiunit responses to the sight of human grasping action, which were arranged by channel configuration. As in **(B)**, the multiunits on the bottom two channels on the second shank strongly responded to the movie.

Before searching for mirror neurons in the frontal cortex, we first performed an electrophysiological mapping of the cortical regions around the STS to carry out *in vivo* imaging of surface connection (Ichinohe et al., 2012) between the frontal cortex and the temporal cortex. Although there is no sulcus in the frontal cortex of the common marmosets, STS in the temporal cortex is one of the clear and rare landmark sulci. In addition, previous studies showed that cells in STS of macaque monkeys and humans represent others' action (Perrett et al., 1989; Gallese et al., 1996; Barraclough et al., 2009; Nelissen et al., 2011), and indeed the posterior part of STS of the common marmosets also contain cells that respond to others' action (Suzuki et al., 2015), which was considered to form mirror neurons in the frontal cortex. By exposing STS as the landmark after craniotomy and duratomy, we conducted multiunit recording under an anesthetic condition using linear array multicontact (32-channels) electrodes and investigated the responses of cells to others' actions, including reaching and grasping a piece of food. We found areas that contained multiunits that strongly responded to others' actions in the posterior part of STS; thus, into these areas we injected a fluorescent retrograde tracer, namely, cholera-toxin b subunit conjugated with Alexa555 (CTB-Alexa555; Figures 1, 2). After 1 week, we were able to identify *in vivo* the connected region in the ventrolateral frontal cortex as labeled spots of a retrograde tracer under a fluorescence stereomicroscope (Figure 1B). In a subsequent histological examination, after all the experiments were finished, each of every three consecutive sections was stained by immuno-histochemistry of CTB, Nissl substrates, or myelin. The latter two series of the stained sections were used to identify brain areas. We found that the injection site in STS located in a ventral part of the fundus of the superior temporal area (FSTv), and the labeled cells were distributed in the ventrolateral frontal cortex including area 6V which is comparable to macaque F4/5 (Burman et al., 2008; Paxinos et al., 2012) where mirror neurons were found, area 8A and area 12L/45 (Figure 1C).

### Mirror neurons in the ventrolateral frontal cortex and STS

To search for mirror neurons, we implanted each of two linear array multicontact (32-channels) electrodes into fluorescently bright spots in the ventrolateral frontal cortex including area 6V and in STS injection sites, which were histologically confirmed later (Figure 1C) (Burman et al., 2008; Paxinos et al., 2012). After waiting for the animal to recover (about 2 days), we conducted multiunit recording under an awake condition. We trained the animal to sit in a primate chair with its head fixed. There were two trial conditions. In the first observation condition, the animal observed an experimenter's action. The experimenter performed one of the eight grasping action types in front of the primate chair with a tray placed just in front of the chair (Materials and Methods). In the second execution condition, we let the animal grasp a piece of banana or bun on the tray.

Individual multiunits were considered to be mirror neurons when their responses were significantly evoked when the animal was observing the experimenter's grasping action (at least in one of eight observation conditions; *t*-test with Bonferroni correction, *p* < 0.05/8) and when the animal itself was performing a grasping action (execution condition; *t*-test, *p* < 0.05), compared with the preceding baseline activity.

**Figure 3:**
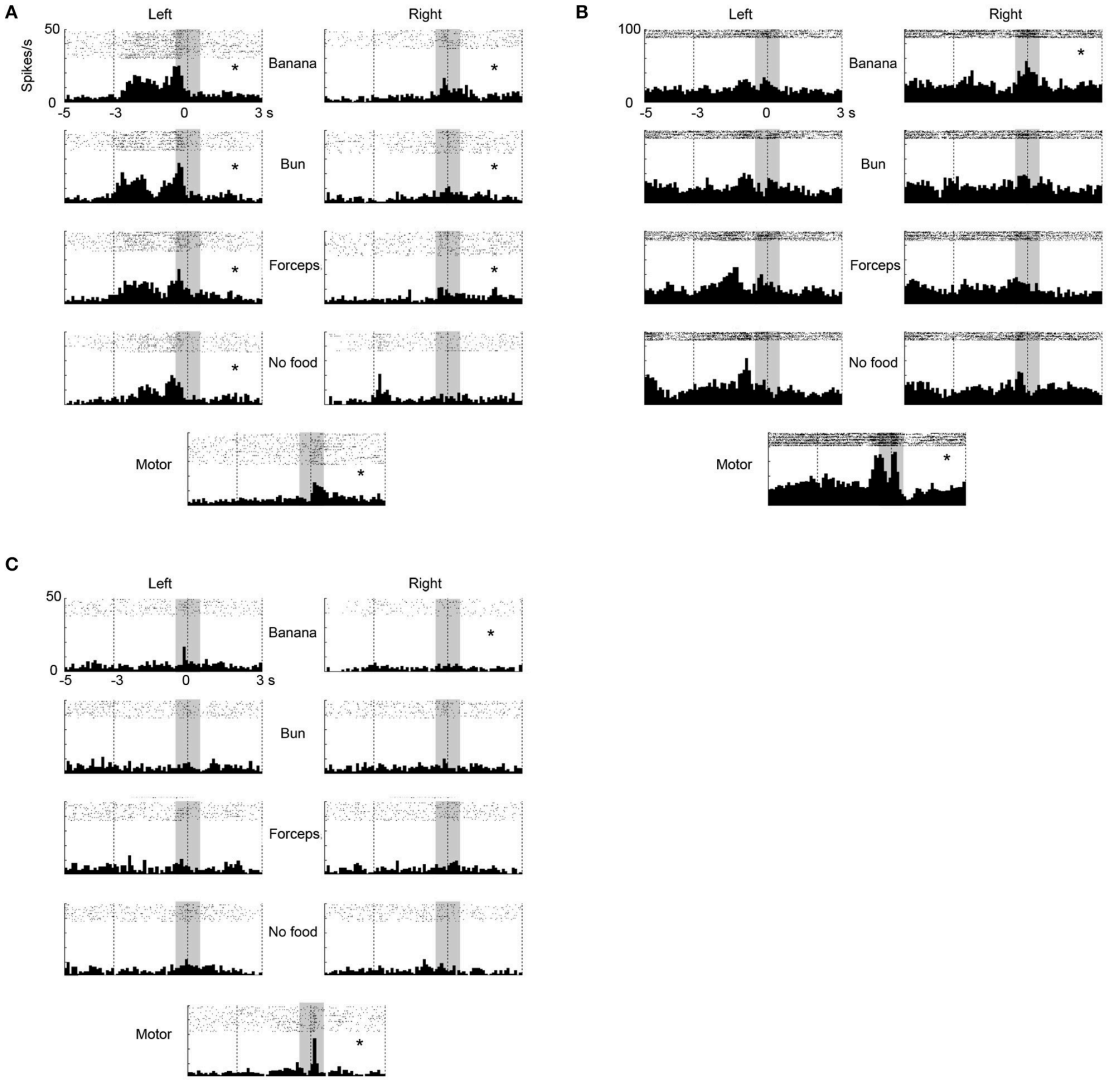
**Three examples of multiunit responses (A–C) with mirror neuron properties under observation and execution conditions in the ventrolateral frontal cortex**. Rasters and peristimulus time histograms were aligned to the touch of food at time = 0. The bin width for the peristimulus time histogram was 100 ms. The left (right) column indicated the multiunit responses when an experimenter grasped food from the left (right) side of the animal. The first to fourth rows indicate the multiunit responses when an experimenter grasped (1) a piece of banana with his/her hand, (2) a piece of bun with his/her hand, (3) a piece of banana with a pair of forceps, and (4) he/she mimed to reach and grasp as if there was a piece of food, respectively. The bottom indicates the multiunit responses when the animal itself grasped a piece of banana or bun. The responses in the shaded area were used for statistical analysis. ^*^p < 0.05/8 under observation condition, *p* < 0.05 under execution condition (paired-*t* test).

In the ventrolateral frontal cortex, 27 (*N* = 6, 14, and 7 from 3 animals, respectively) out of 96 multiunits (recorded from 32 electrodes from each animal) met these mirror neuron criteria. We show here three examples of the responses of mirror neurons to others' action (Figure 3). All showed strong responses both while the animal was observing others grasping for the food and while the animal itself was executing the food grasping, with variations in their response profiles. For example, Figure 3A shows a multiunit that responded when an experimenter grasped a piece of food from the left side of the animal, in general, with a peak observed just before the experimenter touched the food (left columns). On the other hand, a peak was observed just after the animal itself grasped a piece of food (bottom). Figure 3B shows another multiunit that responded when an experimenter grasped a piece of banana with a peak observed at around the time the banana was touched (left and right columns). When the animal grasped a piece of food, two peaks appeared just before and at around the time the food was touched (bottom). Figure 3C shows a third multiunit that responded both when an experimenter grasped a piece of banana, and when the animal grasped a piece of food, with its peak observed at around the time the food was touched.

**Figure 4:**
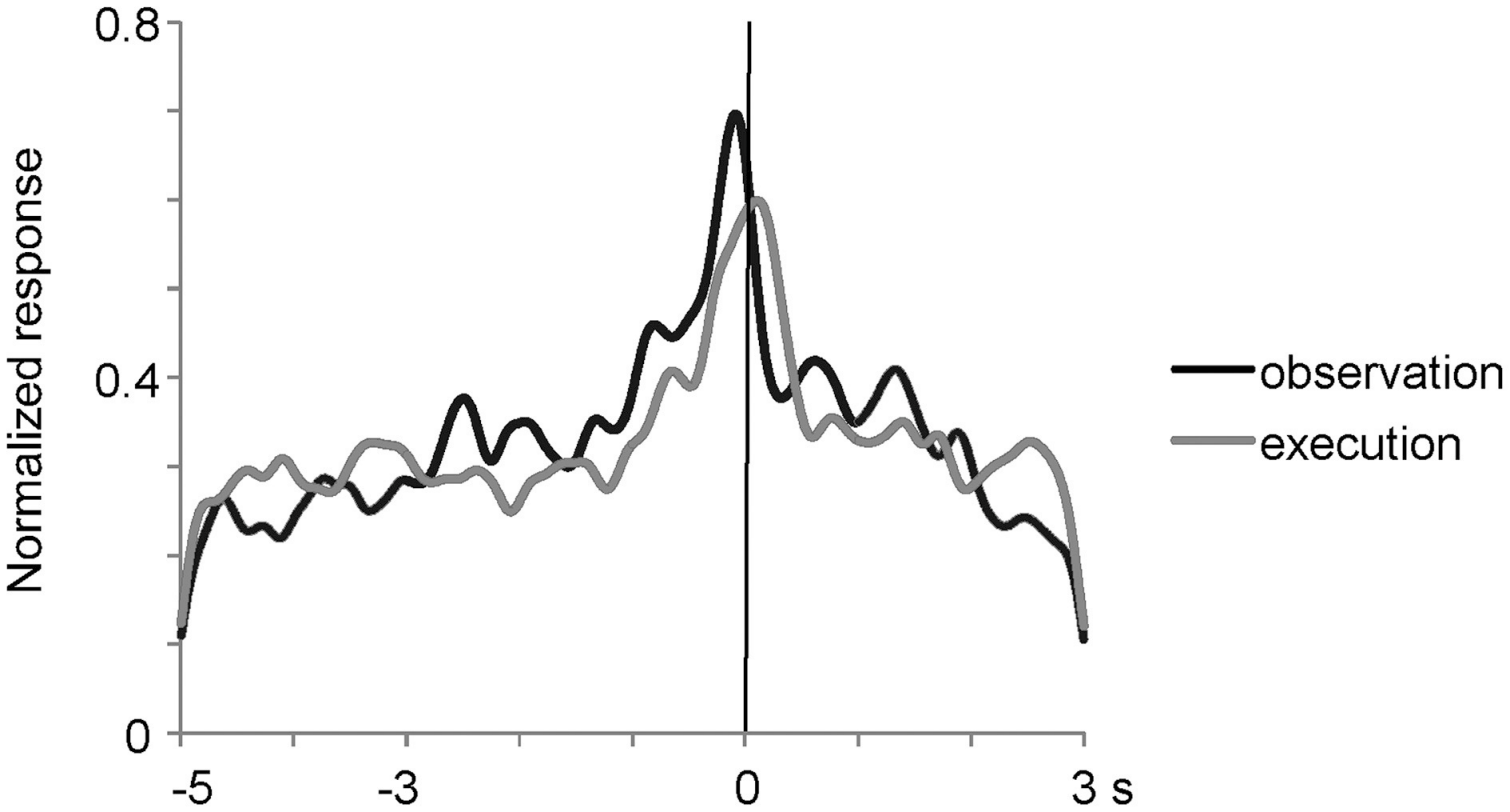
**Time course of normalized activity of neuronal population for 27 mirror neurons in the ventrolateral frontal cortex**. The responses were aligned at the moment when the experimenter or animal touched the food and were averaged after normalizing the responses by the peak responses. Black and gray lines indicate the responses when an experimenter grasped a piece of food under the most preferred grasping action condition and when the animal itself grasped a piece of food, respectively.

To analyze the effect of reaching directions and grasping action types under the observation condition, we applied Two-way factorial ANOVA (with reaching direction and grasping action type as factors) to the responses of the 27 multiunits that met the mirror neuron criteria. Two-thirds (18/27) of them showed either a main effect or interaction. A significant main effect (*p* < 0.05) of reaching direction and grasping action type was observed in 33% (9/27) and 44% (12/27) of the multiunits, respectively. A significant interaction (*p* < 0.05) between the two factors was relatively rare and found in 7% (2/27) of the multiunits. Thus, a significant number of the mirror neurons represented the behavioral manner of others' action as well. The 27 mirror neurons had the activity time course shown in Figure 4, which was aligned at the moment when the experimenter or the animal touched the food. The responses were averaged after normalizing the responses by the peak responses of the individual mirror neurons. The magnitude of responses gradually increased and reached the peak at around the time the food was touched under both observation and execution conditions.

**Figure 5:**
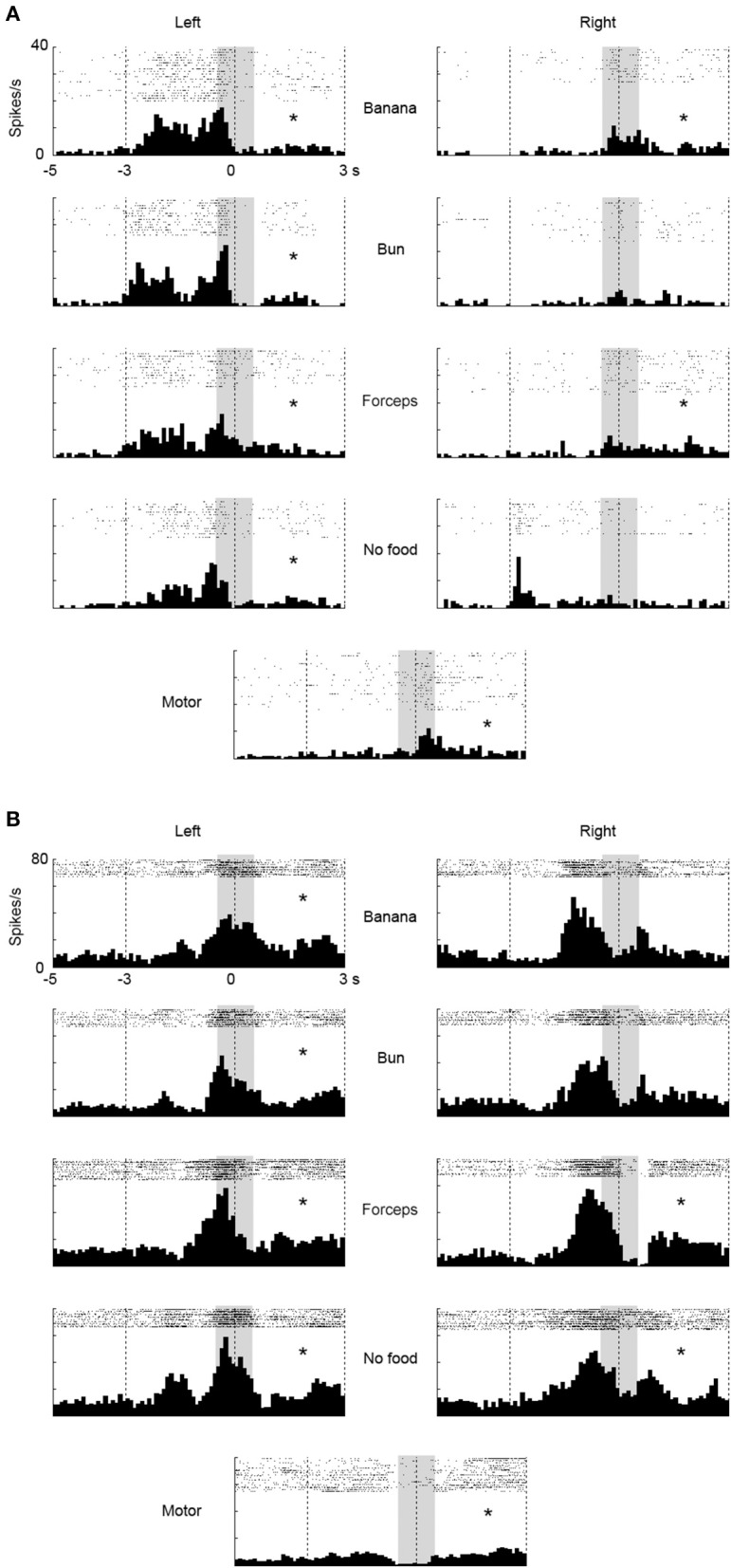
**Two examples of single-unit responses under observation and execution conditions in the ventrolateral frontal cortex**. Display formats were the same as those in Figure 3. **(A)** Single-unit responses that satisfied the mirror neuron criteria. **(B)** Magnitude of single-unit responses that significantly increased under the observation condition, but decreased under the execution condition. ^*^p < 0.05∕8 under observation condition, *p* < 0.05 under execution condition (paired-*t* test).

We investigated a possibility that the multiunits with mirror neuron properties contained two types of single units, one with visual-dominant responses and the other with motor-dominant responses. We sorted single units from the multiunit data in the ventrolateral frontal cortex off-line. Thirty single units (*N* = 11, 4, and 15 from 3 animals) were isolated and 9 (*N* = 4, 1, and 4) satisfied the mirror neuron criteria (Figure 5A). Among the 30 single units, the responses of two single units significantly increased under the observation condition, but decreased under the execution condition (Figure 5B), and those of one single unit decreased under both observation and execution conditions. Among the multiunits and single units that showed either significant visual or motor responses, 34 and 41% satisfied the mirror neuron criteria, respectively (6/32, 14/23, and 7/24 for multiunits and 4/7, 1/4, and 4/11 for single units from 3 animals). Thus, multiunits with mirror neuron properties we found involved single units with the mirror neuron properties.

**Figure 6:**
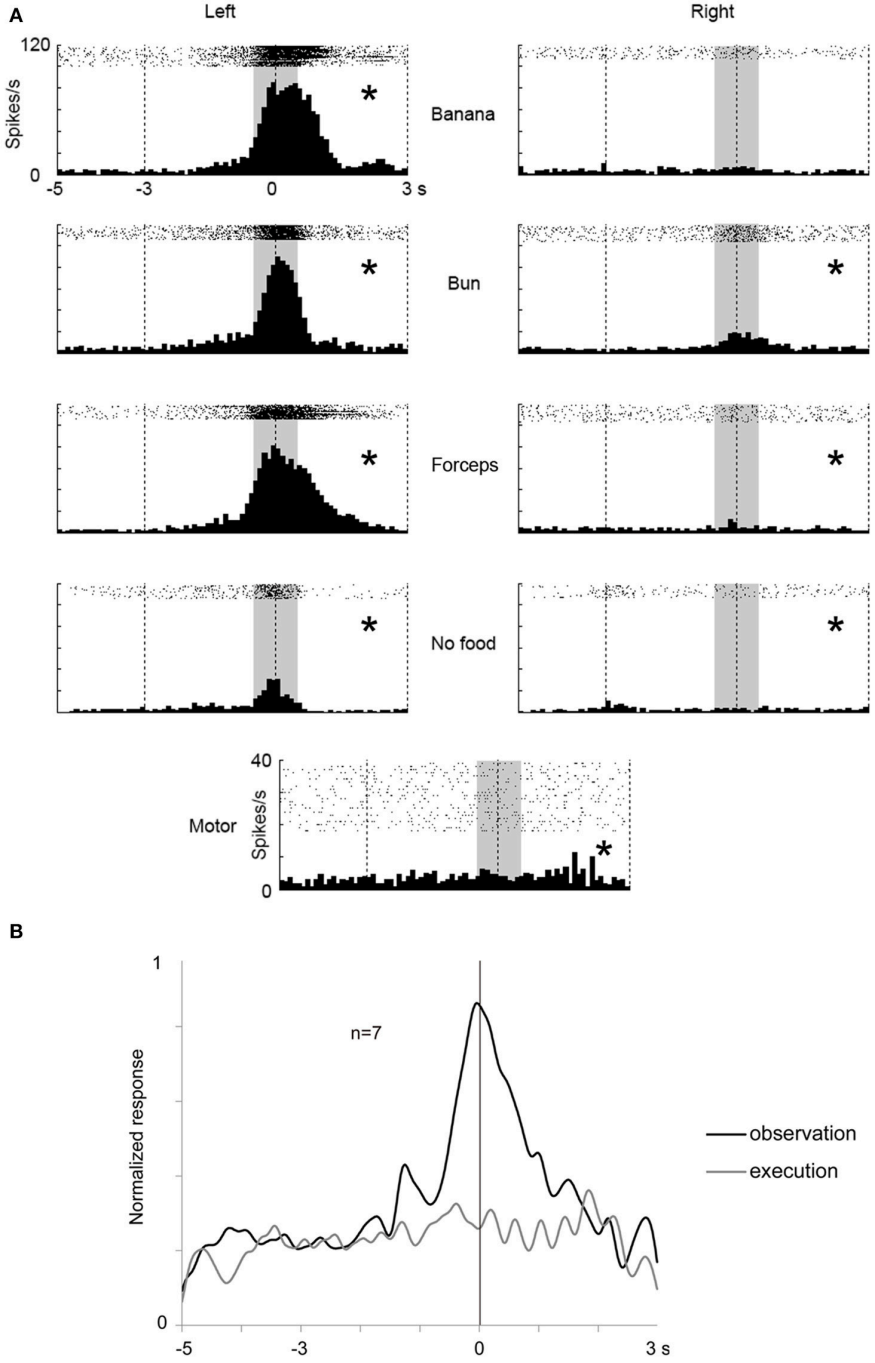
**An example of a mirror neuron in STS and time course of the population activity for seven mirror neurons**. **(A)** Rasters and peristimulus time histograms of an example of STS mirror neuron responses. Display formats are the same as in Figure 3. **(B)** Time course of the normalized activity of neuronal population for seven mirror neurons in STS. Display formats are the same as in Figure 4. ^*^p < 0.05∕8 under observation condition, *p* < 0.05 under execution condition (paired-*t* test).

In STS, we found seven mirror neurons using 32 recorded channels in one animal (see Materials and Methods). A multiunit shown in Figure 6 strongly responded while the animal was observing the experimenter grasping a piece of food from its left side with the response peaking when the experimenter touched the food and slightly, but significantly, responded while the animal itself was grasping the food. As in the case of the frontal cortex, we applied Two-way factorial ANOVA [with reaching direction and grasping action type (see above or “Materials and Methods”) as factors] to the responses of the seven STS mirror neurons. The reaching direction and action type were observed as significant main factors (*p* < 0.05) in 100% (7/7) and 43% (3/7) of the multiunits, respectively. A significant interaction (*p* < 0.05) was found in 57% (4/7) of the multiunits. For the STS mirror neurons, the response magnitude tended to be much larger for the observation than for the execution of grasping action (Figure 6B). The peak of the response for the execution of grasping action was also unclear, while that for the observation was distinctly located at around the time the food was touched. Thus, we found a small number of “mirror neurons” in STS and the magnitude of their responses to the self-action was obviously smaller than those in the ventrolateral frontal cortex.

### Comparison between mirror neurons and visual-dominant cells in the ventrolateral frontal cortex and STS

**Figure 7:**
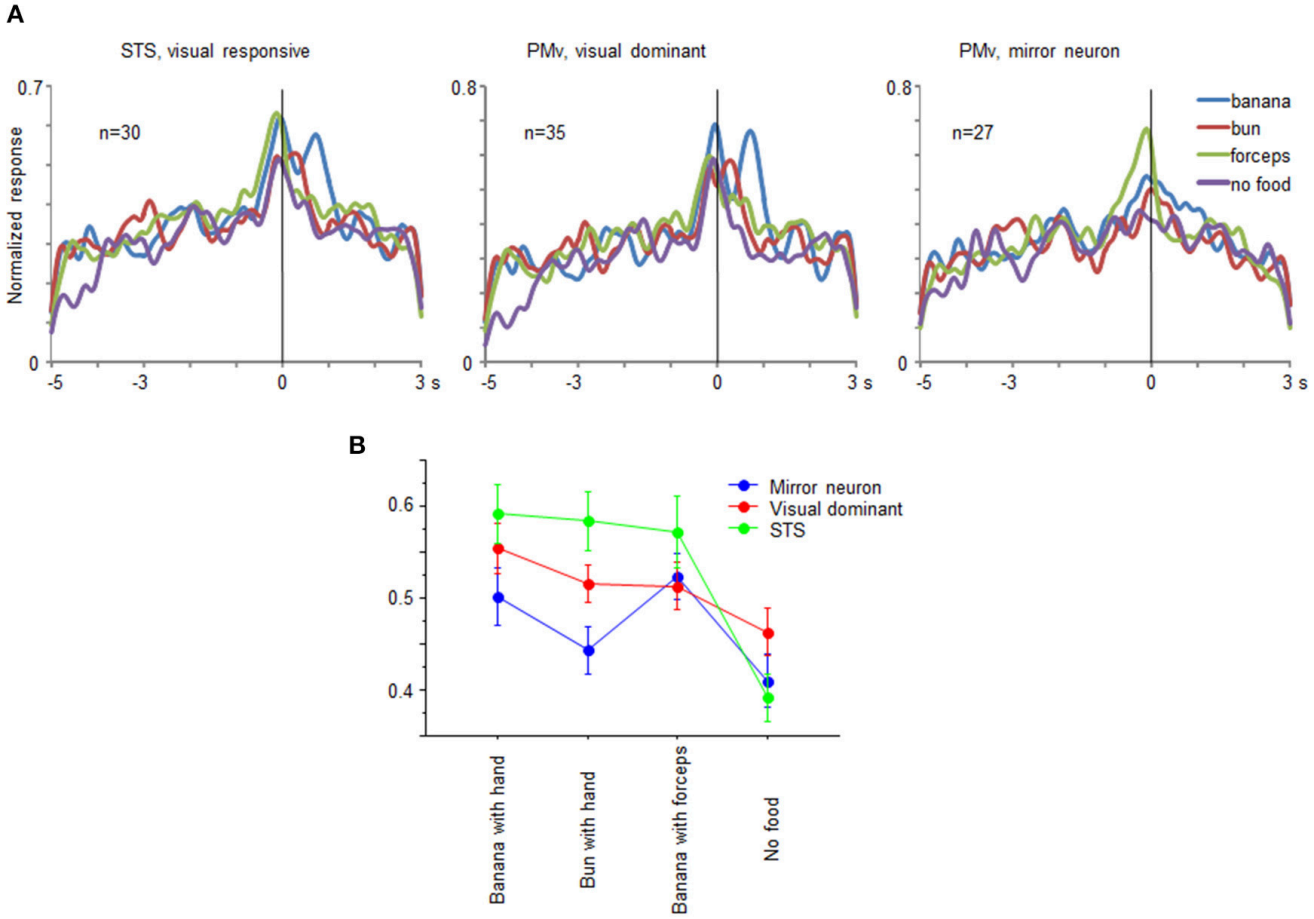
**Comparison of visual-responsive cells in STS, visual-dominant cells in the ventrolateral frontal cortex, and mirror neurons in the ventrolateral frontal cortex**. **(A)** Time course of normalized activity of neuronal population for 30 visual-responsive multiunits in STS, 35 visual-dominant multiunits in the ventrolateral frontal cortex, and 27 mirror neurons in the ventrolateral frontal cortex. Normalized responses of the multiunits for the preferred reaching direction when an experimenter grasped a piece of banana with his/her hand (blue), a piece of bun with his/her hand (red), a piece of banana with a pair of forceps (green), and he/she mimed to reach and grasp as if there was a piece of food (purple). The responses were aligned at the moment when the experimenter or animal touched the food. **(B)** Response magnitudes of visual-responsive cells in STS (green), visual-dominant cells in the ventrolateral frontal cortex (red), and mirror neurons in the ventrolateral frontal cortex (blue) for each grasping action type. Error bars indicate standard error of the mean.

From the recorded regions, besides the mirror neurons, we also found multiunits that responded only under the observation conditions in both the ventrolateral frontal cortex and STS. The multiunits were classified into three groups: mirror neurons in the ventrolateral frontal cortex, visual-dominant cells in the ventrolateral frontal cortex, and visual-responsive cells in STS. We combined the mirror neurons and the visual-dominant cells in STS into one group as the visual-responsive cells, because the motor-related responses were very weak. Multiunits were considered to be visual-dominant cells when these significantly responded while observing at least one of the eight grasping actions (*t*-test with Bonferroni correction, *p* < 0.05/8) but not while executing a grasping action. Figure 7A shows the time course of the activity of a neuronal population for the 30 visual-responsive cells in STS, 35 visual-dominant cells in the ventrolateral frontal cortex, and 27 mirror neurons in the ventrolateral frontal cortex. The responses of the multiunits to each grasping action for the preferred reaching direction were aligned at the moment when the food was touched, normalized by the maximum magnitude of their responses, and averaged across the multiunits for each group. Interestingly, the time-course profile was similar for all three groups; the magnitude of responses gradually increased and reached a peak at the time the food was touched. This similarity of visual responses might be transmitted partly by a the ventrolateral frontal cortex intrinsic connection or a projection from the ventrolateral frontal cortex to STS. Two-way factorial ANOVA (with multiunit groups and grasping action type as factors, Figure 7B) was applied to the responses at around the time the food was touched, and no significant main effect of multiunit groups was shown (*p* = 0.065). However, there was a significant interaction between multiunit groups and grasping action type (*p* = 0.015). The difference between no food condition and the other food conditions was largest in the visual-responsive cells in STS than in the other two groups in the ventrolateral frontal cortex (*p* = 0.0049; One-way ANOVA). Thus, the representation of the grasping action type was different in detail among STS, mirror neurons, and non-mirror neurons in the ventrolateral frontal cortex. Interestingly, STS cells were more sensitive to the reaching goal.

## Discussion

In this study, we found mirror neurons in the ventrolateral frontal cortex of a common marmoset that responded both when performing a grasping action and when observing the experimenter performing a grasping action. This is the first study that showed mirror neurons in a nonhuman primate other than macaque monkeys. We propose that the evolution of mirror neurons responsive to grasping action can be traced to a common ancestor of Old and New World monkeys that are presumed to have a motor repertory such as reaching, grasping, and manipulation actions with hands (Cartmill, 1974; Bloch and Boyer, 2002; Stepniewska et al., 2005), although songbirds are phylogenetically most ancient species where mirror neurons are discovered in forebrain (Prather et al., 2008; Keller and Hahnloser, 2009; Giret et al., 2014).

In this study, we combined electrophysiology with *in vivo* surface connection imaging. First we determined the STS region responsive to the sight of others' action. Second, we visualized the anatomical connections between the STS region and the ventrolateral frontal region using *in vivo* surface connection imaging method. Finally, we conducted unit recordings from the two connected regions. Using this procedure, we were able to search target cells, i.e., mirror neurons, in the frontal cortex efficiently. Otherwise it might be necessary to spend long time to conduct unit recording across the large frontal regions without referring landmarks in marmosets that have no sulcus in the frontal cortex. Another advantage is to enable simultaneous unit recording from two anatomically connected regions with a confirmation *in vivo*. There was a potential disadvantage with this procedure. The regions in which first unit recording was conducted and the anatomical tracers were injected could be damaged owing to several penetration of electrodes and a glass pipet. Indeed, we failed to record from STS of the second and third animals, resulting small samples in the injection site. Also, because the electrodes were chronically implanted after visualizing the anatomical connection, we conducted the unit recording only in one session or in fixed recording site for each awake animal. Although we used 32-channel multicontact electrodes, electrode with a larger number of channels or with movable device would be more preferable to increase the sample size.

There is no direct anatomical connection between the posterior STS and PMv in macaque monkeys (Matelli et al., 1986; Seltzer and Pandya, 1989). Recently, the connection of the caudolateral frontal cortex in common marmosets, however, is investigated using retrograde tracer (Burman et al., 2015) and they find that area 6V, a PMv homolog, receives inputs from FST and the inferior temporal area weakly. In our study, a retrograde tracer injection into area FST labeled cells in the ventrolateral frontal cortex including area 6V (Figure 1C), suggesting reciprocal anatomical connections between the posterior STS and area 6V in marmosets. The labeled cells were also distributed in areas 8A and 12L/45 consistent with the previous study (Reser et al., 2013). A recent study showed that the cells in areas 8A, 12L/45, and 6V of common marmosets were involved in vocal-signal processing and vocal-motor production (Miller et al., 2015). The anatomical input from extrastriate cortex indicates that the ventrolateral frontal cortex also contributes to visual information processing for social communication.

The mirror neurons in the ventrolateral frontal cortex of marmosets found in this study have properties common to those in PMv of macaque monkeys (Rizzolatti and Craighero, 2004). In the macaque, observation of a grasping action strongly activates the mirror neurons in PMv, with their activity peaking at around the time the food is touched. Mimicking the grasping action, the type of effector to grasp, and the action direction significantly modulated the mirror neuron responses (Gallese et al., 1996). These properties were also commonly observed in the mirror neurons in the ventrolateral frontal cortex of marmoset.

It is suggested that mirror neurons in songbirds are neural substrates for imitative motor learning by transforming from perception into similarly complex action (Giret et al., 2014). The temporal profile difference between sensory response and motor response of mirror neurons of songbirds may be accounted for by Hebbian learning theory. In this study, the peak of the responses might be slightly earlier when observing the experimenter performing a grasping action than when performing a grasping action (Figure 5), which was inconsistent with the temporal profile difference found in mirror neurons of songbirds. However, it was difficult to compare whether the two peaks were significantly different because the profiles of hand motion for marmosets (execution) and for human (observation) were different and the time resolution of monitor system was poor (33.3 ms). This issue might be resolved by using actor marmosets for the observation condition and a monitor system with better time resolution.

Mirror neurons in macaque monkeys are subdivided into “strictly congruent” and “broadly congruent” depending on the relationships between the visual features of the observed action they responded to and the motor response they code (Gallese et al., 1996). When their preferences for the observed action and executed action match in terms of means of action (e.g., precision grip or power grip), they are classified as “strictly congruent,” and the other mirror neurons are classified as “broadly congruent.” One-third of the mirror neurons are “strictly congruent” and two-thirds are “broadly congruent” in macaque monkeys (Gallese et al., 1996). All the mirror neurons recorded in this study should be inherently classified as “broadly congruent,” because, in this study, the experimenter used precision grip, and common marmosets exclusively use power grip to manipulate objects (van Schaik et al., 1999).

A combination between a grasping hand and the target to grasp is essential in activating mirror neurons in PMv and some cells in STS of macaque monkeys (Perrett et al., 1989; Gallese et al., 1996; Barraclough et al., 2009; Nelissen et al., 2011). Consistent with this, we showed that the absence of the target to grasp decreased the magnitude of responses of the mirror neurons in the ventrolateral frontal cortex and the visual-responsive cells in STS of common marmosets. Furthermore, by directly comparing between the responses of mirror neurons and non-mirror neurons in the ventrolateral frontal cortex and visual-responsive cells in STS, we found that mirror and non-mirror neurons in the ventrolateral frontal cortex were not as sensitive to the presence of the target to grasp as STS cells. This suggests that the mirror neurons in the ventrolateral frontal cortex and STS cells play different roles in understanding others' action.

Although we found only a few mirror neurons in STS, we determined that the magnitude of their responses to the execution of grasping action were smaller than those to the observation of grasping action. Even under the execution condition, theoretically, the animals' own hands with which they grasp food could elicit a visual response in STS cells. Hietanen and Perrett (1993) showed that STS cells in macaque monkeys do not respond to self-induced movement of their own hands. It was suggested that the inhibition of visual response to self-induced hand movement might be used to discriminate an animal's own actions from those of the others (Keysers and Perrett, 2004). This inhibition might arise from the ventrolateral frontal cortex (mirror neurons or visual- dominant neurons) and transmitted to STS through a direct connection, which this study has shown.

Common marmosets live together as a large family, with the mother, the father, and older siblings. Almost all family members engage in parenting behaviors including carrying, grooming, protecting, and feeding infants (Ferrari, 1992; Rothe et al., 1993). Moreover, they imitate a novel action demonstrated by a conspecific (Voelkl and Huber, 2000, 2007). Future research on mirror neurons in the common marmoset and their role in unique social behaviors will further deepen our understanding of the functions of mirror neurons.

## Funding

This work was supported by an Intramural Research Grant (Grant No. 23-7) for Neurological and Psychiatric Disorders from NCNP, Grant-in-Aid for Scientific Research (C) (26430031), a Funding Program for World-Leading Innovative R&D on Science and Technology (FIRST Program), a Grant-in-Aid on Innovative Areas, “Shitsukan,” and “Adolescent Mind and Self-Regulation” of MEXT, and the program for Brain Mapping by Integrated Neurotechnologies for Disease Studies (Brain/MINDS) from AMED, Japan.

### Conflict of interest statement

The authors declare that the research was conducted in the absence of any commercial or financial relationships that could be construed as a potential conflict of interest.
